# Oxymatrine Modulation of TLR3 Signaling: A Dual-Action Mechanism for H9N2 Avian Influenza Virus Defense and Immune Regulation

**DOI:** 10.3390/molecules29091945

**Published:** 2024-04-24

**Authors:** Yan Zhi, Xinping Zhao, Zhenyi Liu, Guoyu Shen, Taiming Zhang, Tao Zhang, Ge Hu

**Affiliations:** College of Animal Science and Technology, Beijing University of Agriculture, Beijing 102206, China; bjzhiyan@sina.com (Y.Z.); 202130311012@bua.edu.cn (X.Z.); liuzylynn@163.com (Z.L.); zytgzyyx0723@163.com (G.S.); 202330311012@bua.edu.cn (T.Z.); zhangtao8008@126.com (T.Z.)

**Keywords:** H9N2 Avian Influenza Virus (AIV), pulmonary microvascular endothelial cells (PMVECs), Oxymatrine (OMT), antiviral mechanisms, TLR3 signaling pathway, immune modulation

## Abstract

In our research, we explored a natural substance called Oxymatrine, found in a traditional Chinese medicinal plant, to fight against a common bird flu virus known as H9N2. This virus not only affects birds but can also pose a threat to human health. We focused on how this natural compound can help in stopping the virus from spreading in cells that line the lungs of birds and potentially humans. Our findings show that Oxymatrine can both directly block the virus and boost the body’s immune response against it. This dual-action mechanism is particularly interesting because it indicates that Oxymatrine might be a useful tool in developing new ways to prevent and treat this type of bird flu. Understanding how Oxymatrine works against the H9N2 virus could lead to safer and more natural ways to combat viral infections in animals and humans, contributing to the health and well-being of society. The H9N2 Avian Influenza Virus (AIV) is a persistent health threat because of its rapid mutation rate and the limited efficacy of vaccines, underscoring the urgent need for innovative therapies. This study investigated the H9N2 AIV antiviral properties of Oxymatrine (OMT), a compound derived from traditional Chinese medicine, particularly focusing on its interaction with pulmonary microvascular endothelial cells (PMVECs). Employing an array of in vitro assays, including 50% tissue culture infectious dose, Cell Counting Kit-8, reverse transcription-quantitative polymerase chain reaction, enzyme-linked immunosorbent assay, and Western blot, we systematically elucidated the multifaceted effects of OMT. OMT dose-dependently inhibited critical antiviral proteins (PKR and Mx1) and modulated the expression of type I interferons and key cytokines (IFN-α, IFN-β, IL-6, and TNF-α), thereby affecting TLR3 signaling and its downstream elements (NF-κB and IRF-3). OMT’s antiviral efficacy extended beyond TLR3-mediated responses, suggesting its potential as a versatile antiviral agent. This study not only contributes to the growing body of research on the use of natural compounds as antiviral agents but also underscores the importance of further investigating the broader application of OMT for combating viral infections.

## 1. Introduction

Influenza viruses, which are classified into four types (A, B, C, and D), pose significant challenges to public health because of their varied nucleoproteins and M proteins [1,2,3]. Type A influenza, particularly the H9N2 avian influenza strain, has garnered attention within the scientific community because of its widespread prevalence and the potential threat it poses to humans [4,5]. Despite being classified as low pathogenicity, H9N2 is known for its rapid transmission among poultry, contributing to considerable economic losses and carrying a potential risk of zoonotic transmission [6,7]. The risk it poses is compounded by its robust mutagenic capabilities and gene recombination potential, leading to significant concerns in both veterinary and human medicine and highlighting the critical limitations of current vaccine strategies against rapidly evolving influenza viruses [8,9].

The intricate interaction between the Avian Influenza Virus (AIV) and the host immune system, primarily through the Toll-like receptor 3 (TLR3) pathway, is central to inflammatory response development and immune regulation following infection [10]. Previous studies have illustrated the upregulation of TLR3 and IFN-β expression in avian tissues after infection with avian influenza, illustrating the significant role of TLR3 in bolstering host defense mechanisms [11]. In pulmonary microvascular endothelial cells (PMVECs), which play a pivotal role in maintaining the vascular barrier and modulating the immune response [12], H9N2 triggers a complex network of pathogen-associated molecular pattern (PAMP) recognition [13] with TLR3 serving as a key receptor for detecting viral double-stranded RNA (dsRNA). This activation initiates a cascade of signaling events leading to the production of type I interferons and inflammatory cytokines, kickstarting the innate immune response which is a critical first line of defense against viral infections [14,15].

Traditional Chinese medicine (TCM) offers a distinct perspective on antiviral therapy, emphasizing treatments that are less likely to induce drug resistance. Oxymatrine (OMT), a quinolizidine alkaloid extracted from the roots of *Sophora flavescens* Aiton and *Sophora tonkinensis* Gagnep (Fabaceae) [16,17], exhibits a broad spectrum of biological activities [18], including hepatoprotection, anti-tumorigenesis, anti-inflammation, and antibacterial effects [19,20,21]. Its capacity to modulate the immune response and inhibit viral replication of hepatitis B virus (HBV) in transgenic mice underscores its potential as an immunomodulatory agent [22]. Its documented impacts on type A influenza virus replication and inflammation modulation through the TLR4, p38 MAPK, and NF-κB pathways have provided a foundation for its exploration as an antiviral agent against H9N2 avian influenza [23]. However, the specific mechanisms by which OMT interacts with the TLR3 signaling pathway in avian influenza infection remain largely unexplored, marking a significant gap in the current understanding of its antiviral efficacy.

This study aims to elucidate the antiviral effects of OMT against the H9N2 AIV with a focus on the TLR3 signaling pathway. RNA interference (RNAi) was used in primary rat PMVECs to investigate the antiviral efficacy of OMT against H9N2 AIV and to explore its broader application as a modern antiviral therapeutic derived from traditional Chinese medicine. Acknowledging potential limitations inherent to in vitro studies, this investigation paves the way for future in vivo research to comprehensively assess the therapeutic potential and safety profile of OMT. By integrating traditional medicine insights with contemporary virological research, this study contributes to the urgent search for novel antiviral strategies to fight rapidly mutating influenza viruses, offering promising directions for future scientific exploration and therapeutic innovation.

## 2. Results

### 2.1. Observation and Immunofluorescent Identification of PMVECs

**Figure 1:**
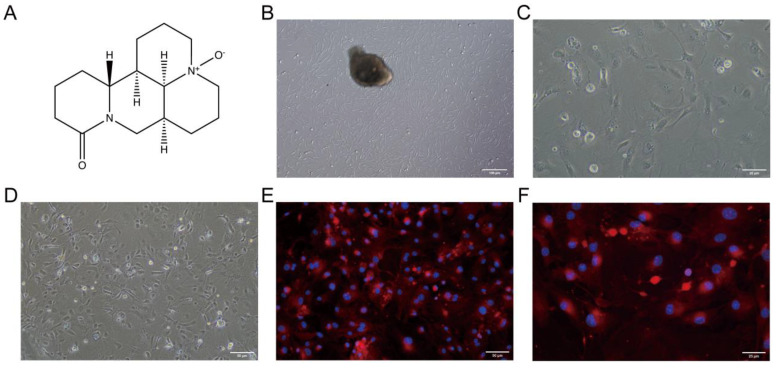
Chemical structure of Oxymatrine (OMT) and morphological observation of PMVECs pre- and post-purification with CD31 immunofluorescence Identification. (**A**) Chemical structure of Oxymatrine (OMT). (**B**) Illustrates the growth state of primary isolated pulmonary microvascular endothelial cells (PMVECs) at 60 h before purification captured under phase-contrast microscopy. Scale bar = 100 μm, indicating the initial confluence and morphology of cells. (**C**,**D**) Display PMVECs post-purification, observed under phase-contrast microscopy at 200× (**C**) and 400× (**D**) magnification. These panels detail the refined growth state and cellular morphology post-purification with scale bars of 25 μm and 50 μm, respectively, showcasing enhanced cell purity and uniformity. (**E**,**F**) Demonstrate immunofluorescence identification of the CD31 antigen on PMVECs with merged images at 200× (**E**) and 400× (**F**) magnification. CD31 is marked by red fluorescence, while nuclei are counterstained blue with DAPI, affirming the endothelial nature of the cells. The distinct CD31 expression, combined with the observed cellular morphology, underscores the endothelial identity of PMVECs, establishing their appropriateness for the ensuing experiments. Scale bars are set at 50 μm and 25 μm for (**E**) and (**F**), respectively.

The morphological changes of primary isolated PMVECs were monitored over time. Initial migration from the tissue block periphery was observed approximately 6 h after culture initiation, gradually escalating over time (Supplementary Figure S3A–D). After 60 h, cells achieved significant density, allowing for the removal of the tissue block; the block displayed a cobblestone morphology characterized by an interlocked arrangement, though some hematopoietic cells were also present (Figure 1B). Following the media change, a notable transition to a spindled morphology was observed with increasing passage number, indicating continuous cell proliferation (Figure 1C,D).

Further validation of PMVEC identity was achieved through immunofluorescence analysis. Staining for CD31, a hallmark endothelial marker, resulted in pronounced red fluorescence, while nuclei were counterstained blue with DAPI (Supplementary Figure S4). Merged imaging confirmed that the majority of the observed cells were PMVECs (Figure 1E,F), establishing their suitability for use in subsequent experiments to investigate the effects of H9N2 AIV infection and OMT treatment.

### 2.2. H9N2 AIV Infectivity in PMVECs Measured Using TCID50

To determine the infectivity of H9N2 AIV in PMVECs, we propagated the virus in MDCK cells using gradient dilutions and closely monitored the induced pathological changes in PMVECs. This facilitated the calculation of the TCID50 for H9N2 AIV, which was determined to be 10^−4.8^ per 0.1 mL based on the Reed–Muench method (Table 1). Initiation with a 10^−2^ dilution resulted in a virus titer of 10^−3^/mL within PMVECs, underscoring the potent infectivity of H9N2 in this cell type. There was a clear pattern in H9N2 AIV’s ability to infect PMVECs with a 100% infection rate observed at higher viral concentrations; this trend decreased with increasing viral dilution. The TCID50 demonstrated the virus’s potent infectivity and established a foundational understanding for the subsequent OMT treatments.

### 2.3. Cytotoxic Effects of OMT on PMVECs Assessed with the CCK-8 Assay

**Figure 2:**
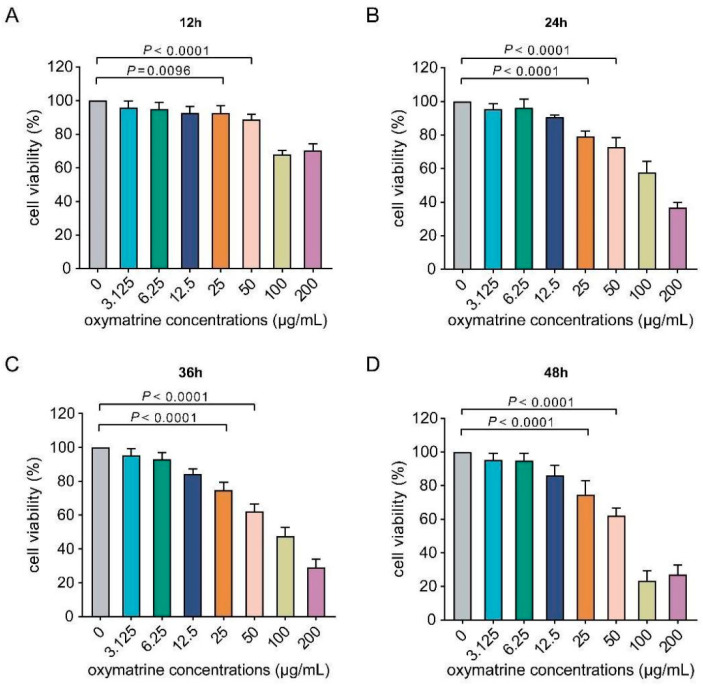
Evaluation of PMVEC viability post-OMT treatment across time. (**A**–**D**) Depict the influence of various Oxymatrine (OMT) concentrations on pulmonary microvascular endothelial cell (PMVEC) viability at different time points: 12 h (**A**), 24 h (**B**), 36 h (**C**), and 48 h (**D**). Cell survival rates were assessed quantitatively through the CCK-8 assay, highlighting the dose- and time-dependent effects of OMT on cell viability. Data represent the mean ± standard deviation (S.D.) from three independent experiments (*n* = 3). Statistical significance was evaluated using non-parametric one-way ANOVA to compare the effects of different OMT concentrations over time.

To comprehensively investigate the cytotoxic effects of OMT on PMVECs, we assessed cell viability across a spectrum of OMT concentrations at intervals of 12, 24, 36, and 48 h. The CCK-8 assessment of the effects of OMT exposure indicated a significant reduction in cell viability at OMT concentrations >12.5 μg/mL (*p* < 0.0001) (Figure 2 and Table 2). This illustrated that OMT exhibited dose-dependent cytotoxicity with higher OMT doses reducing PMVEC survival rates.

In light of these results, screening of a narrow concentration gradient was conducted to pinpoint the drug dosages that would optimize the balance between therapeutic efficacy and cell viability (Figure S5 and Table S3). Based on these results, OMT concentrations of 2.5, 5, and 10 μg/mL were selected for subsequent experiments.

### 2.4. The Influence of OMT on Antiviral Protein Expression in H9N2 AIV-Infected PMVECs

To explore the antiviral potential of OMT in H9N2 AIV-infected PMVECs, we examined the mRNA expression modulation of key antiviral proteins (PKR and Mx1). Using RT-qPCR for precise quantification, we observed an increase in the expression of PKR and Mx1 following H9N2 AIV infection indicative of the cellular response to viral invasion.

**Figure 3:**
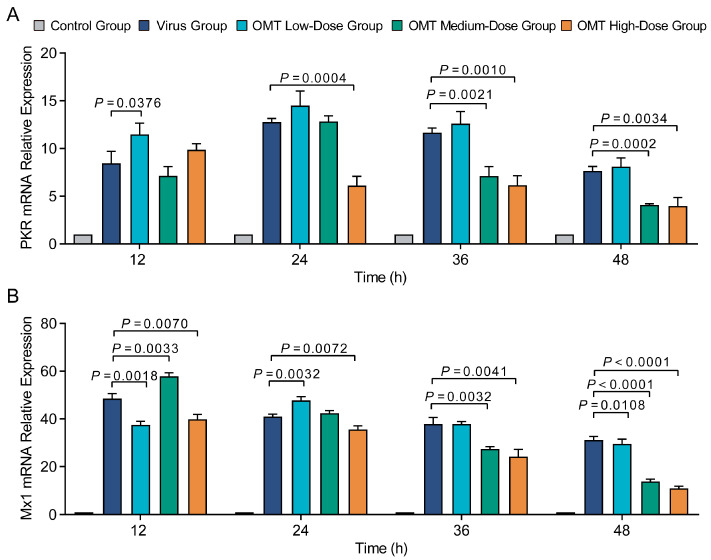
Modulation of antiviral protein mRNA expression in PMVECs by OMT post-H9N2 AIV infection. (**A**,**B**) Display the changes in mRNA expression levels of the antiviral proteins PKR (**A**) and Mx1 (**B**) in PMVECs, following infection with H9N2 AIV and treatment with varying concentrations of Oxymatrine (OMT). These graphs illustrate how OMT treatment influences the antiviral response at the molecular level in cells exposed to H9N2 AIV. Data are presented as mean ± standard deviation (S.D.) from three independent experiments (*n* = 3), underscoring the reproducibility of the observed effects. Statistical analysis was conducted using non-parametric one-way ANOVA to assess the significance of changes in mRNA expression across different OMT dosages.

Treatment with OMT revealed dose-dependent dynamics modulating these antiviral proteins. Treatment with a low dose of OMT increased PKR mRNA levels (*p* < 0.05), suggesting an initial bolstering of the antiviral response. As the OMT dosage increased, a progressive decrease in PKR mRNA expression was observed (*p* < 0.01) (Figure 3A). This pattern suggests that while low-dose OMT may stimulate antiviral defense mechanisms, higher doses could potentially mitigate this response.

Similarly, Mx1 mRNA expression exhibited a dose-responsive increase at low and medium OMT dosages (*p* < 0.05), aligning with the expected antiviral reinforcement based on PKR expression patterns; suppression of Mx1 expression was seen with high-dose OMT treatment. By the 48 h mark, a reduction in Mx1 mRNA levels was seen across all OMT concentrations (*p* < 0.01), irrespective of OMT dosage (Figure 3B). Overall, the results indicated interactions between treatment duration, OMT dosage, and antiviral protein expression modulation.

### 2.5. Regulatory Effects of OMT on the Expression of IFN-α, IFN-β, IL-6, and TNF-α in PMVECs Infected with H9N2 AIV

**Figure 4:**
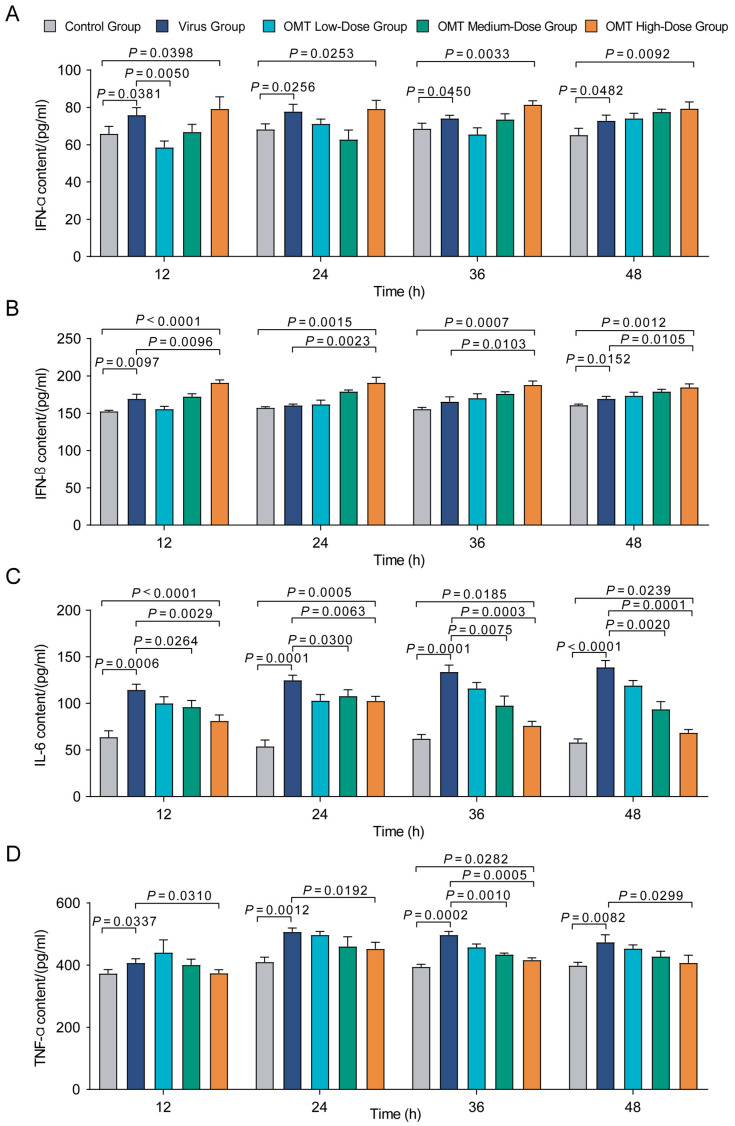
Cytokine level changes in PMVECs following H9N2 AIV infection and OMT treatment. (**A**–**D**) Demonstrate the quantitative changes in cytokine levels—IFN-α (**A**), IFN-β (**B**), IL-6 (**C**), and TNF-α (**D**)—among PMVECs following exposure to H9N2 AIV and subsequent treatment with Oxymatrine (OMT) at various dosages and time points. These panels highlight the modulatory effect of OMT on key cytokines integral to the immune response against viral infection. Results are expressed as mean ± standard deviation (S.D.) for three replicates (*n* = 3), reflecting the consistency of OMT’s impact across experiments. Statistical significance of cytokine level variations between treatment groups was assessed using non-parametric one-way ANOVA.

In our investigation into the immunomodulatory effects of OMT on H9N2 AIV-infected PMVECs, we observed significant alterations in the expression of critical cytokines. Post-infection treatment with varying OMT doses revealed a distinct modulation pattern of IFN-α, IFN-β, IL-6, and TNF-α levels, indicating effects shaping the antiviral immune response. Early observations (12 to 24 h) found a significant surge in IFN-α levels in both virus and high-dose OMT groups compared with controls (*p* < 0.05), suggesting an intensified antiviral state (Figure 4A). In contrast, low-dose OMT attenuated IFN-α expression relative to the virus-only group (*p* = 0.005), highlighting a dose-dependent immunological influence.

Analysis of IFN-β expression, particularly at 24 and 36 h post-treatment, showed that medium and high doses of OMT not only elevated IFN-β beyond control levels but also beyond those observed in the virus group (*p* < 0.01) (Figure 4B). IL-6 levels across all OMT treatment groups consistently fell below those in the virus group with the reduction being most notable at the 12 h interval (*p* < 0.0001), indicating a mitigated inflammatory response (Figure 4C). Similarly, TNF-α levels were decreased across all OMT dosages at 36 h post-infection compared with the virus group (*p* < 0.05), further confirming OMT’s anti-inflammatory capacity (Figure 4D).

Overall, OMT was observed to have dose- and time-dependent regulatory effects on cytokine expression in H9N2 AIV-infected PMVECs, indicating its dual role in enhancing antiviral pathways while tempering inflammatory responses.

### 2.6. Effects of OMT on mRNA and Protein Expression Levels of NF-κB, IRF-3, and TLR3 in H9N2 AIV-Infected PMVECs

**Figure 5:**
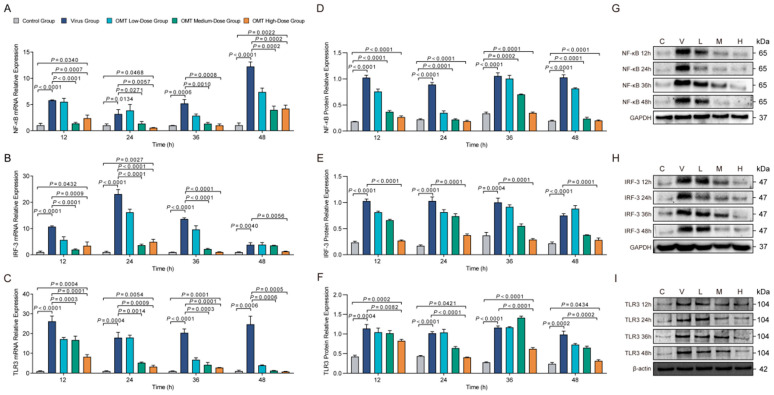
OMT modulates NF-κB, IRF-3, and TLR3 expression in H9N2 AIV-infected PMVECs. (**A**–**C**) Illustrate the changes in mRNA expression of NF-κB (**A**), IRF-3 (**B**), and TLR3 (**C**) in PMVECs subjected to different OMT treatments post-H9N2 AIV infection, showcasing the transcriptional modulation over time. (**D**–**F**) Display the alterations in protein expression of NF-κB (**D**), IRF-3 (**E**), and TLR3 (**F**) under similar experimental conditions, highlighting OMT’s influence at the protein level. (**G**–**I**) Western blot images corresponding to NF-κB (**G**), IRF-3 (**H**), and TLR3 (**I**), providing visual evidence of the protein expression changes. Each experiment was conducted in triplicate, with data presented as mean ± standard deviation (S.D.) for three independent experiments (*n* = 3). Statistical analysis of the expression levels was performed using non-parametric one-way ANOVA to determine the significance of differences observed across treatment groups over the investigated time points.

Significant modulations in both mRNA and protein expression levels of key antiviral signaling molecules (NF-κB, IRF-3, and TLR3) were seen through comprehensive RT-qPCR and Western blot analyses. In H9N2 AIV-infected PMVECs treated with OMT at varied dosages, we observed a distinctive pattern; within the first 24 h, upregulation in NF-κB and IRF-3 mRNA expression was seen in cells treated with low-dose OMT, pointing to an enhanced initial antiviral response (*p* < 0.01) (Figure 5A–C). Conversely, treatment with medium and high doses of OMT resulted in a decrease in mRNA expression, suggesting dose-dependent attenuation across all time points examined (*p* < 0.01). TLR3 mRNA expression also followed this pattern with significant elevation seen in the low-dose OMT treatment group by 48 h, whereas medium and high doses resulted in consistent decreases in expression (*p* < 0.01).

Protein expression analyses using Western blotting reinforced the dose-dependent effects of OMT on the antiviral landscape within PMVECs. Elevated levels of TLR3 and NF-κB were consistently observed across all examined intervals in both the virus-infected and OMT-treated cohorts (*p* < 0.05), indicating sustained activation of antiviral signaling. High-dose OMT treatment significantly reduced TLR3 protein levels at specific intervals, while NF-κB protein expression, despite remaining elevated under viral challenge, was notably mitigated by all dosages of OMT (*p* < 0.0001). Additionally, IRF-3 protein levels decreased across all time points in the high-dose OMT group compared with treated virus-only cells (*p* = 0.0001), highlighting OMT’s intricate regulation of the immune response machinery of PMVECs infected with H9N2 AIV. These results revealed that OMT has both time- and dose-dependent dynamics that significantly influence the cellular antiviral response it induces.

### 2.7. Effects of OMT on mRNA and Protein Expression of NF-κB and IRF-3 Post-TLR3 Silencing in H9N2 AIV-Infected PMVECs

**Figure 6:**
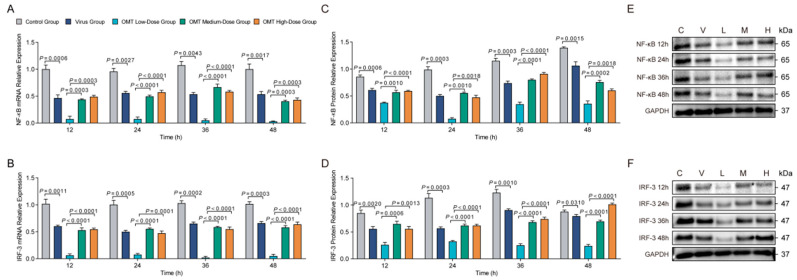
Effects of OMT on NF-κB and IRF-3 post-TLR3 silencing in H9N2 AIV-infected PMVECs. (**A**,**B**) Showcase the alterations in mRNA expression levels of NF-κB (**A**) and IRF-3 (**B**) in PMVECs, following TLR3 silencing and subsequent H9N2 AIV infection under varying OMT treatments. These panels elucidate the transcriptional changes induced by OMT in the absence of TLR3 signaling. (**C**,**D**) Detail the changes in protein expression of NF-κB (**C**) and IRF-3 (**D**) across different OMT treatment groups over time, highlighting how OMT modulates these key regulatory proteins in the context of TLR3 silenced PMVECs infected with H9N2 AIV. (**E**,**F**) Present the Western blot images for NF-κB (**E**) and IRF-3 (**F**), providing visual confirmation of the protein expression variations. Data represent the mean ± standard deviation (S.D.) from three replicates (*n* = 3), ensuring the reliability of the results. Statistical analyses to assess the impact of OMT on NF-κB and IRF-3 expression levels were performed using non-parametric one-way ANOVA, identifying significant differences across treatment groups and over time.

Post-TLR3 silencing, a decrease in NF-κB mRNA expression was observed across all examined time points following H9N2 AIV infection (*p* < 0.005), underscoring its pivotal role in regulating NF-κB transcription in response to viral infection (Figure 6A). The administration of a medium dose of OMT (5 µg/mL) significantly elevated NF-κB mRNA levels at all time points (*p* < 0.0005), revealing OMT’s capacity to counterbalance the effects of TLR3 silencing on NF-κB expression. The employment of different si-RNAs for TLR3 silencing resulted in variations in NF-κB mRNA responses at 36 and 48 h post-infection (*p* < 0.0005), suggesting specificity in the si-RNA-mediated modulation of NF-κB. Similarly, IRF-3 mRNA levels experienced a significant reduction post-TLR3 silencing at all time points following H9N2 AIV infection (*p* < 0.005), aligning with the trends observed for NF-κB and highlighting the interconnected regulatory mechanisms governing IRF-3 expression (Figure 6B). The application of a medium dose of OMT post-TLR3 silencing increased IRF-3 mRNA levels at all time points (*p* < 0.0001), indicating OMT’s modulatory influence extends to IRF-3. The effect of different si-RNA types on IRF-3 mRNA levels further emphasized the complex interplay between TLR3 silencing and the regulation of IRF-3 regulation (*p* < 0.05).

Western blot analyses corroborated these mRNA trends at the protein level with NF-κB and IRF-3 protein expression reduced in the TLR3 RNAi + H9N2 AIV-infected groups (*p* < 0.01; Figure 6C–F). Medium-dose OMT treatment post-TLR3 silencing counteracted these declines and also introduced variances in NF-κB and IRF-3 protein levels dependent on the si-RNA type used (*p* < 0.01), though transient effects were seen for NF-κB. These findings collectively highlighted the nuanced role of OMT in modulating NF-κB and IRF-3 expression in the backdrop of TLR3 silencing in virally infected cells.

## 3. Discussion

H9N2 AIV predominantly targets the respiratory tract by infecting PMVECs, which are crucial for forming a vascular barrier and modulating the immune response. Despite the virus’s relatively low pathogenicity, its rapid mutation and efficient transmission rates, compounded by inadequate preventative measures, pose significant challenges to current vaccine strategies, necessitating the exploration of alternative therapies [24]. Our study examined the therapeutic potential of OMT, a quintessential component of traditional Chinese medicine, showcasing its dual-action mechanism modulating both cellular responses to H9N2 AIV infection and immune response regulation. The observed inhibition of PKR and Mx1 mRNA expression after H9N2 AIV infection underscores OMT’s potential to interfere with host–virus interactions at the molecular level [25,26], possibly through modulation of TLR3 signaling [27]. Additionally, its capacity to upregulate type I interferons (IFN-α and IFN-β), pivotal in orchestrating innate immune responses, aligns with results from emerging research on the antiviral properties of natural compounds [28,29,30] and reinforces the therapeutic promise of OMT against H9N2 AIV.

The role of the TLR3 signaling pathway in the immune response to H9N2 AIV infection and the subsequent impact of OMT treatment are compelling aspects of the therapeutic potential of this compound. Silencing TLR3 resulted in a reduction in the expression of NF-κB and IRF-3 at both the mRNA and protein levels, underscoring the critical function of TLR3 in mediating antiviral immune responses [31]. Despite TLR3 silencing, the antiviral activity of OMT persisted, suggesting that it engages multiple different pathways when exerting its effects. This indicates that OMT’s antiviral efficacy extends beyond the TLR3 signaling axis, potentially broadening its therapeutic utility against viruses that might evade or suppress TLR3-mediated responses because of mutations or other infection mechanisms [32]; additionally, it posits the existence of a polypharmacological landscape where OMT acts on various signaling pathways, possibly in parallel or synergistically, to mount a comprehensive defense against H9N2 AIV [33,34,35]. This multifaceted approach to antiviral defense underscores the complexity of host–virus interactions and highlights the potential for OMT to offer a robust and adaptable therapeutic strategy against rapidly evolving viral threats. The modulation of NF-κB and IRF-3 expression by OMT post-TLR3 silencing aligns with the broader understanding of TLR3′s involvement in viral infections and immune regulation. These findings suggest that OMT’s influence on these transcription factors could mitigate excessive inflammatory reactions while enhancing antiviral defenses, offering insights into the delicate balance required in antiviral therapy [34]. However, it is crucial to recognize that immune signaling pathways form intricate networks with interactions that extend beyond the scope of TLR3; other pathways and molecules, including TLR4, TLR7, MAPK, TNF-α, RIG-I, and MITA may also play roles in the antiviral response modulated by OMT [33,34]. The interplay between these pathways suggests a complex signaling tapestry that is possibly influenced by OMT, necessitating further research to unravel the comprehensive signaling networks that it affects in the context of viral infections.

Despite its findings, our study is not without limitations. The primary reliance on in vitro models, while foundational, means that further in vivo studies are required to ascertain the clinical relevance and therapeutic efficacy of OMT against H9N2 AIV. Future research should aim to validate these findings through comprehensive animal model studies, carefully selecting models that mimic the human response to H9N2 AIV infection [35]. Such studies will be critical in evaluating the safety, optimal dosing, and pharmacokinetics of OMT, addressing potential challenges such as bioavailability and adverse effects.

In conclusion, OMT presents a promising avenue for antiviral therapy against H9N2 AIV with its dual-action mechanism offering both direct antiviral activities and immune system modulation. Future application of this promising therapeutic agent will require a concerted effort to transition from in vitro findings to in vivo validation as well as clinical trials. Collaborations with clinical research teams and regulatory bodies will be essential to navigate the complexities of bringing a traditional Chinese medicine component like OMT into the realm of clinically approved antiviral therapies. Despite the challenges ahead, the potential of OMT as a multifaceted therapeutic agent against H9N2 AIV infection remains promising, meriting further investigation and development.

## 4. Materials and Methods

### 4.1. Cell Lines and Viruses

We used primary Madin–Darby Canine Kidney (MDCK) cells and PMVECs as foundational cell lines. MDCK cells, maintained within our laboratory, were cultured in Dulbecco’s Modified Eagle’s Medium (DMEM; BioWhittaker, Walkersville, MD, USA) enriched with 10% fetal bovine serum (FBS; Gibco-BRL, New York, NY, USA) and a 2% penicillin/streptomycin antibiotic/antimycotic mixture (GIBCO-BRL, New York, NY, USA). PMVECs, derived from 7–9-day-old rats using the tissue block explant method [36], received supplementation with 15 μg/mL endothelial cell growth supplement (ECGS; Millipore, Billerica, MA, USA) in DMEM throughout the isolation process. Subsequently, PMVECs were also cultured in DMEM supplemented with 10% FBS and 2% penicillin/streptomycin, with all cell lines incubated at 37 °C in a humidified atmosphere containing 5% CO_2_.

The low pathogenicity AIV (LPAIV) H9N2 subtype was used, sourced from the laboratory at China Agricultural University, with GenBank accession numbers FJ499463–FJ499470. The propagation of viruses was performed in MDCKs, PMVECs, or 11-day-old specific pathogen-free (SPF) embryonated chicken eggs, employing previously outlined methodologies [37,38].

### 4.2. Immunofluorescence Characterization of PMVECs

To verify the identity of PMVECs, we adapted and refined the immunofluorescence method outlined by Kovacs-Kàsa, ensuring specificity in our cell characterization [39]. Following the culture, PMVECs designated for staining underwent fixation with 4% paraformaldehyde and were permeabilized with 0.5% Triton X-100 for 20 min, setting the stage for antibody binding. Blocking with normal goat serum for 30 min minimized non-specific interactions, preceding overnight incubation at 4 °C with a primary antibody against CD31, a definitive marker for endothelial cells, at a 1:100 dilution. This step was followed by an hour’s incubation at room temperature with a Cy3-conjugated secondary antibody, also at a 1:100 dilution, to enhance visibility under fluorescence microscopy. Subsequent to staining with DAPI for 5 min to delineate nuclei, slides were mounted using an anti-fade mounting medium. Each staining step was interspersed with three PBST washes for 5 min each to ensure clarity. Imaging was performed under fluorescence microscopy with appropriately calibrated imaging parameters to capture representative images.

### 4.3. siRNA-Mediated TLR3 Silencing in PMVECs

To elucidate the role of TLR3 in the immune response against H9N2 AIV in PMVECs, we employed a targeted gene silencing approach using small interfering RNA (siRNA). The siRNA sequences (see Supplementary Table S1), designed to specifically target the rat TLR3 gene, were synthesized by Beijing Qinkai Biotechnology Co., Ltd. (Beijing, China). Sequences were selected based on their alignment with the TLR3 gene in the NCBI database, with specificity confirmed through BLAST analysis to ensure exclusivity to the rat TLR3 gene and mitigate potential off-target effects on other genes.

Before initiating siRNA transfection, we conducted preliminary screening to determine the safety concentrations for both the siRNA and the transfection reagent (see Supplementary Figures S1 and S2). The selection of optimal siRNA candidates involved time-course studies, concentration assessments, and analyses of sequence efficacy, culminating in the identification of sequences that achieved a target interference efficiency between 60% to 70% [40].

### 4.4. OMT Characterization and Cytotoxicity Assessment

OMT [16,17] was procured from Shanghai Yuan-ye Biotechnology Co., Ltd. (Shanghai, China) The chemical structure of OMT is delineated in Figure 1A. To assess cytotoxicity, PMVECs were initially seeded in a 96-well polypropylene plate at a density of 10,000 cells per mL, using 100 µL of medium per well, and incubated at 37 °C in an atmosphere containing 5% CO_2_. Upon reaching 80% confluence, PMVECs were exposed to varying concentrations of OMT (2.5, 5, and 10 µg/mL) diluted in phenol-red free DMEM, using DMSO as the solvent to maintain a consistent and non-toxic concentration across treatments. Each OMT concentration was tested in sextuplicate, while control wells were treated with DMEM containing only DMSO. To mitigate edge evaporation effects during the experiment, the perimeter wells of the plate were filled with PBS.

Cell viability post-OMT exposure was evaluated at intervals of 12, 24, 36, and 48 h. This involved washing the cells with 200 µL PBS, which was followed by the addition of 10 µL of CCK-8 reagent (Beijing Solar-bio Science & Technology Co., Ltd., Beijing, China) to each well. After incubating for 2–4 h to facilitate formazan crystal formation, absorbance was measured at 450 nm, using a reference wavelength between 600 and 650 nm. Cell viability was quantified using the following formula:

$$Cell\;Viability(\%)=\frac{Absorbance\;of\;control\;sample}{Absorbance\;of\;test\;sample}\times 100\%$$

### 4.5. Determination of H9N2 TCID_50_

To accurately quantify the viral titers capable of infecting 50% of the MDCK cell population, we employed the Reed and Muench method [41]. MDCK cells were prepared at a density of 10,000 cells/mL, seeded into a 96-well plate, and cultivated at 37 °C in a 5% CO_2_ atmosphere until reaching approximately 80% confluence, at which point the cells were washed with PBS. The H9N2 AIV was serially diluted in tenfold increments ranging from 10^−1^ to 10^−10^ which were applied to the MDCK monolayers. Following a 1 h adsorption period at 37 °C in 5% CO_2_, the inoculum was replaced with a maintenance medium containing 2% DMEM. The presence of a cytopathic effect (CPE) exceeding 70% over a 72 h observation period was used to identify infected wells.

In parallel, PMVECs achieving over 90% confluence in 6-well plates were exposed to various doses of the virus (1X, 50X, and 100X TCID_50_) to determine the optimal infectious dose based on the extent of CPE observed.

### 4.6. Experimental Grouping and Treatment Methods

We aimed to elucidate the impact of OMT on cytokine expression and key signaling pathways, including TLR3, NF-κB, and IRF-3, in PMVECs following infection with H9N2 AIV. The experimental design was structured into two main objectives with corresponding groups and treatment protocols. Initially, to assess the influence of OMT on cytokine expression in PMVECs post-H9N2 AIV infection, cells were divided into several groups: a control group (uninfected), an H9N2 AIV-infected group without OMT treatment, and H9N2 AIV-infected groups treated with OMT at varying concentrations (2.5, 5, and 10 µg/mL). Samples were collected at predetermined intervals of 12, 24, 36, and 48 h post-infection with each time point represented by triplicate samples to ensure robust data collection.

To evaluate the role of OMT in TLR3-silenced PMVECs infected with H9N2 AIV, experimental groups included an H9N2 AIV-infected control group without TLR3 silencing, a group with TLR3 RNAi followed by H9N2 AIV infection, and groups where TLR3-silenced PMVECs were infected with H9N2 AIV and treated with a medium-dose of OMT (5 µg/mL). This setup also incorporated two variations of TLR3 RNAi combined with medium-dose OMT treatment post-H9N2 AIV infection. Sampling for this segment of the study mirrored the previous arrangement, with collections at 12, 24, 36, and 48 h intervals post-infection, with each time point supported by triplicate samples.

### 4.7. ELISA Determination of Cytokine Levels

Following experimental treatments, cytokine levels (IFN-β, IFN-α, IL-6, and TNF-α) were quantitatively determined using ELISA, employing commercial kits from ABclonal (Wuhan, China) in strict accordance with the provided instructions [42]. Each cytokine assay was performed according to the manufacturer’s specific instructions, which included distinct incubation times and coating concentrations for the capture antibodies, ensuring measurement accuracy and reliability. Samples were homogenized and centrifuged at 1000 rpm for 10 min at 4 °C to separate the supernatant, which was then assayed for cytokine concentration. The optical density (OD) of each sample was measured at 450 nm with a TECAN F50 spectrophotometer (Mannedorf, Switzerland).

### 4.8. RNA Extraction and Reverse-Transcription Quantitative PCR

After cytokine assessment, RNA was extracted from PMVECs using the TransZol Up kit (Takara, Dalian, China). Extracted RNA were promptly reverse-transcribed to cDNA, which was then stored at −80 °C until further analysis. RT-qPCR was conducted using 2× Hieff UNICON^®^ Universal Blue qPCR Master Mix (Yeasen, Shanghai, China) following a protocol that included UDG activation and an initial denaturation step, followed by 40 amplification cycles, and a final melting curve analysis to ensure specificity. Each sample underwent triplicate analyses to ensure reproducibility and accuracy. β-actin rRNA was used as the endogenous control for the normalization of gene expression with fold changes in target genes calculated via the 2^−ΔΔCT^ method as detailed in prior studies [43]. The genes of interest and corresponding primer sequences employed in this study are listed in Supplementary Table S2 with resultant data analyzed using GraphPad Prism 8.

### 4.9. Western Blot Analysis of Protein Expression in PMVECs

To delineate the protein expression profile in PMVECs in response to H9N2 AIV infection and OMT treatment, Western blot analysis was conducted. PMVECs, seeded in 6-well plates at a density of 10^5^ cells/mL, were cultured to achieve 80–90% confluence. Cells were then subjected to 1 h of H9N2 AIV exposure at 37 °C, while control groups were treated with 2% maintenance medium. After infection, cells underwent treatment with OMT at concentrations of 2.5, 5, or 10 μg/mL. At 12, 24, 36, and 48 h post-infection, proteins were extracted by washing cells with chilled PBS and lysing in cold RIPA:PMSF buffer (100:1) for 20–30 min. After centrifugation at 12,000 rpm for 20 min at 4 °C, supernatants were preserved for subsequent analysis [44].

Protein content was quantified using the BCA assay (Beyotime, Shanghai, China). Electrophoresis was performed on 15% SDS-PAGE, which was followed by protein transfer to membranes at 100 V for 2 h. Membranes were blocked before incubation overnight at 4 °C with primary antibodies targeting TLR3 (DF6415), NF-κB p65 (AF5006), and IRF3 (DF6895) obtained from Jiangsu Qinke Biotechnology Research Center, Suzhou, China. β-actin (20536-1-AP) and GAPDH (10494-1-AP) antibodies, procured from Wuhan Sanying Biotechnology, Wuhan, China, served as loading controls. After incubation with secondary antibodies for 90 min, protein bands were visualized using the Odyssey infrared imaging system (LI-COR Biosciences, Lincoln, NE, USA) and analyzed relative to β-actin or GAPDH expression using ImageJ software (version 1.51j8, National Institutes of Health, Bethesda, MD, USA).

### 4.10. Statistical Analysis

Statistical evaluation of the data was conducted using GraphPad Prism (Version 8.0, San Diego, CA, USA) with results presented as means ± standard deviations. To discern the statistical differences between two independent groups, an unpaired Student’s *t*-test was applied. Comparisons among multiple groups were facilitated by one-way analysis of variance (ANOVA). The intensities of Western blot bands were determined using ImageJ software. A *p*-value < 0.05 was established as the threshold for statistical significance.

## 5. Conclusions

In summary, our study positions Oxymatrine (OMT) as a promising antiviral and immunomodulatory candidate against H9N2 Avian Influenza Virus (AIV). While OMT appears to influence multiple immune pathways, enhancing its antiviral action beyond the TLR3 pathway, it is important to note that our findings are preliminary. Future in vivo studies and clinical trials are crucial to validate OMT’s efficacy and safety comprehensively. Our results serve as an initial step toward exploring OMT’s potential in antiviral therapy, highlighting the need for further research to confirm its therapeutic applicability against viral infections.

## Figures and Tables

**Table 1:** TCID50 determination results of H9N2 AIV.

Virus Dilution	Observed CPE			Cumulative CPE			Infection Rate (%)
	Well Count	CPE Positive Wells	CPE Negative Wells	Cumulative CPE Positive Wells	Cumulative CPE Negative Wells	Proportion of CPE Positive Wells	CPE Positive Rate (%)
10^−1^	8	8	0	33	0	33/33	100
10^−2^	8	8	0	25	0	25/25	100
10^−3^	8	8	0	17	0	17/17	100
10^−4^	8	6	2	11	2	11/13	84.61
10^−5^	8	3	5	5	7	5/12	41.67
10^−6^	8	2	6	2	13	2/15	13.33
10^−7^	8	0	8	0	21	0/21	0

Note: CPE = cytopathic effect.

**Table 2:** Impact of various OMT concentrations on PMVECs survival rate.

		OMT Concentration (μg/mL)	0	3.125	6.25	12.5	25	50	100	200
	Survival Rate (%)									
Time (h)										
12			100.00	95.96	95.03	92.72	92.52	88.77	68.06	70.39
24			100.00	95.36	96.23	90.67	79.12	72.94	57.66	36.79
36			100.00	95.39	93.52	84.81	74.71	62.07	47.47	29.69
48			100.00	95.98	95.06	84.34	73.61	63.61	27.38	27.04

## Data Availability

Data are contained within the article and Supplementary Materials.

## Supplementary Materials

The following supporting information can be downloaded at: https://www.mdpi.com/article/10.3390/molecules29091945/s1. Due to space constraints, any details referred to in the text can be found in the Supplementary Materials.

## Abbreviations

AIV: Avian Influenza Virus; CCK-8, Cell Counting Kit-8; ELISA, enzyme-linked immunosorbent assay; IFN-α, interferon alpha; IFN-β, interferon beta; IL-6, interleukin 6; IRF-3, Interferon Regulatory Factor 3; Mx1, Myxovirus Resistance 1; NF-κB, Nuclear Factor Kappa-Light-Chain-Enhancer of Activated B Cells; OMT, Oxymatrine; PKR, Protein Kinase R; PMVECs, pulmonary microvascular endothelial cells; RNAi, RNA interference; RT-qPCR, Reverse Transcription Quantitative Polymerase Chain Reaction; TCID50, Tissue Culture Infectious Dose 50; TLR3, Toll-like receptor 3; TNF-α, Tumor Necrosis Factor Alpha.
