# Supplementary material for: Oxymatrine Modulation of TLR3 Signaling: A Dual-Action Mechanism for H9N2 Avian Influenza Virus Defense and Immune Regulation
Source: Molecules. 2024 Apr 24;29(9):1945. doi: 10.3390/molecules29091945 (PMC11085666; doi:10.3390/molecules29091945)
Supplement: Supplementary file 1 [file molecules-29-01945-s001.zip › molecules-2941879-supplementary.pdf]

# Supplementary for Oxymatrine Modulation of TLR3 Signaling: A Dual-Action Mechanism for H9N2 Avian Influenza Virus Defense and Immune Regulation

**Yan Zhi †, Xinping Zhao †, Zhenyi Liu, Guoyu Shen, Taiming Zhang, Tao Zhang and Ge Hu ***

College of Animal Science and Technology, Beijing University of Agriculture, Beijing 102206, China; bjzhiyan@sina.com (Y.Z.); 202130311012@bua.edu.cn (X.Z.); liuzylynn@163.com (Z.L.); zytgzyyx0723@163.com (G.S.); 202330311012@bua.edu.cn (T.Z.); zhangtao8008@126.com (T.Z.)

* Correspondence: bnhuge@126.com; Tel.: +86 13693501841

† These authors contributed equally to this work.

**This file includes:**

Tables S1–S3

Figures S1–S5

**Content list**

**Supplementary Tables**

Table S1 ---------------------------------------------------------------------- 1
Table S2 ---------------------------------------------------------------------- 2
Table S3 ---------------------------------------------------------------------- 3

**Supplementary Figures**

Figure S1 ------------------------------------------------------------------------ 4
Figure S2 ------------------------------------------------------------------------ 5
Figure S3 ------------------------------------------------------------------------ 6
Figure S4 ------------------------------------------------------------------------ 7
Figure S5 ------------------------------------------------------------------------ 8

**Table S1. siRNA sequences**

| Group | Target Sequence | siRNA **Sequence** |
|---|---|---|
| TLR3 RNAi | si-TLR3-1-ss | GGUGAGCAGUUCGAAUACATT |
| | si-TLR3-1-as | UGUAUUCGAACUGCUCACCTT |
| | si-TLR3-2-ss | GUUCAAGAACUUAUUCGAATT |
| | si-TLR3-2-as | UUCGAAUAAGUUCUUGAACTT |
| | si-TLR3-3-ss | CAUGGACGAUAAUACUAUATT |
| | si-TLR3-3-as | UAUAGUAUUAUCGUCCAUGTT |
| **Negative Control** | TLR3-negative | siRNA Negative Control |

Note：The aforementioned siRNA sequences, along with the negative control sequence, were subjected to NCBI's BLAST search and showed specificity only to the rat genome, exclusively aligning with the TLR3 sequence, ensuring no off-target effects on other genes.

**Table S2. Quantitative Real-time PCR Primer sequences**

| Genes | Primer Direction | Sequences (5'→3') |
|---|---|---|
| PKR | Forward primer | CCGGATCCAGAAAGTCGGTC |
| | Reverse primer | TTCGCCTCCTGCTTTGATCT |
| Mx1 | Forward primer | CGGAGGATTGGTAAGCCCTCT |
| | Reverse primer | TATCCACAGACTCCATTGCCTG |
| TLR3 | Forward primer | GGTCAGTTTATGAATTCCAGGAGG |
| | Reverse primer | GGGCAGTTTTACTTCCCCGA |
| NF-κB | Forward primer | ATACCACTGTCAACAGATGGCCC |
| | Reverse primer | TTCCATTTGTGACCAACTGAACG |
| IRF-3 | Forward primer | GTATGCGCAGGCTGGATCA |
| | Reverse primer | CAGGTTGACAGGTCTGGCTT |
| β-actin | Forward primer | CCCGCGAGTACAACCTTCTT |
| | Reverse primer | CCATACCCACCATCACACCC |
| NAPDH | Forward primer | CCCCCATGTTTGTGATGGGT |
| | Reverse primer | GCACGATGCATTGCTGACAA |

**Table S3. Effects of different low concentrations of OMT on the survival rate of PMVECs**

| OMT Concentration ($\mu g/ml$) / Survival Rate (%) / Time (h) | 0 | 2.5 | 5 | 7.5 | 10 | 12.5 | 15 |
|---|---|---|---|---|---|---|---|
| 12 | 100.00 | 100.76 | 97.36 | 96.94 | 94.92 | 100.11 | 100.74 |
| 24 | 100.00 | 99.52 | 97.92 | 96.15 | 94.76 | 94.29 | 98.55 |
| 36 | 100.00 | 100.07 | 98.88 | 103.82 | 95.78 | 92.53 | 88.35 |
| 48 | 100.00 | 99.29 | 100.50 | 97.85 | 94.77 | 91.27 | 87.93 |

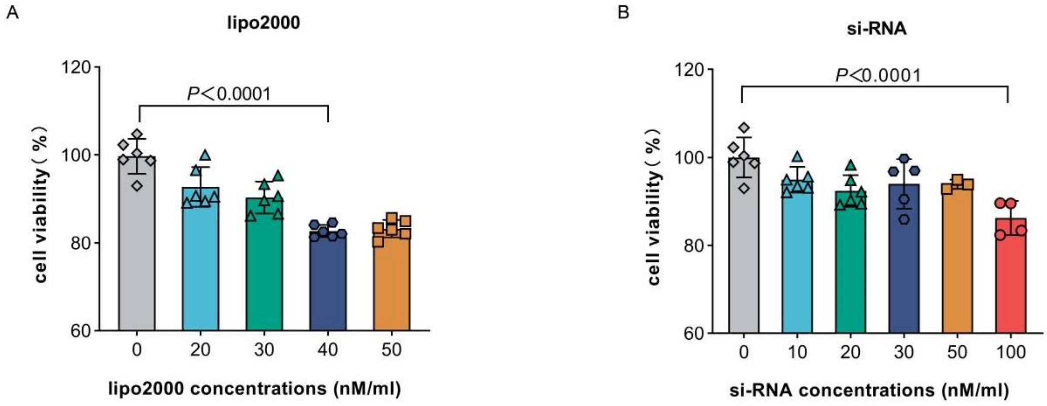


**Figure S1. Safe concentration of siRNA and transfection reagent.**

(A) Determination of the safe concentration for transfection reagent.

(B) Selection of the optimal concentration for siRNA.

Experiments were performed as three biologically independent experiments, and the mean ± S.D. (n = 6) is shown. P values were determined by non-parametric one-way ANOVA.

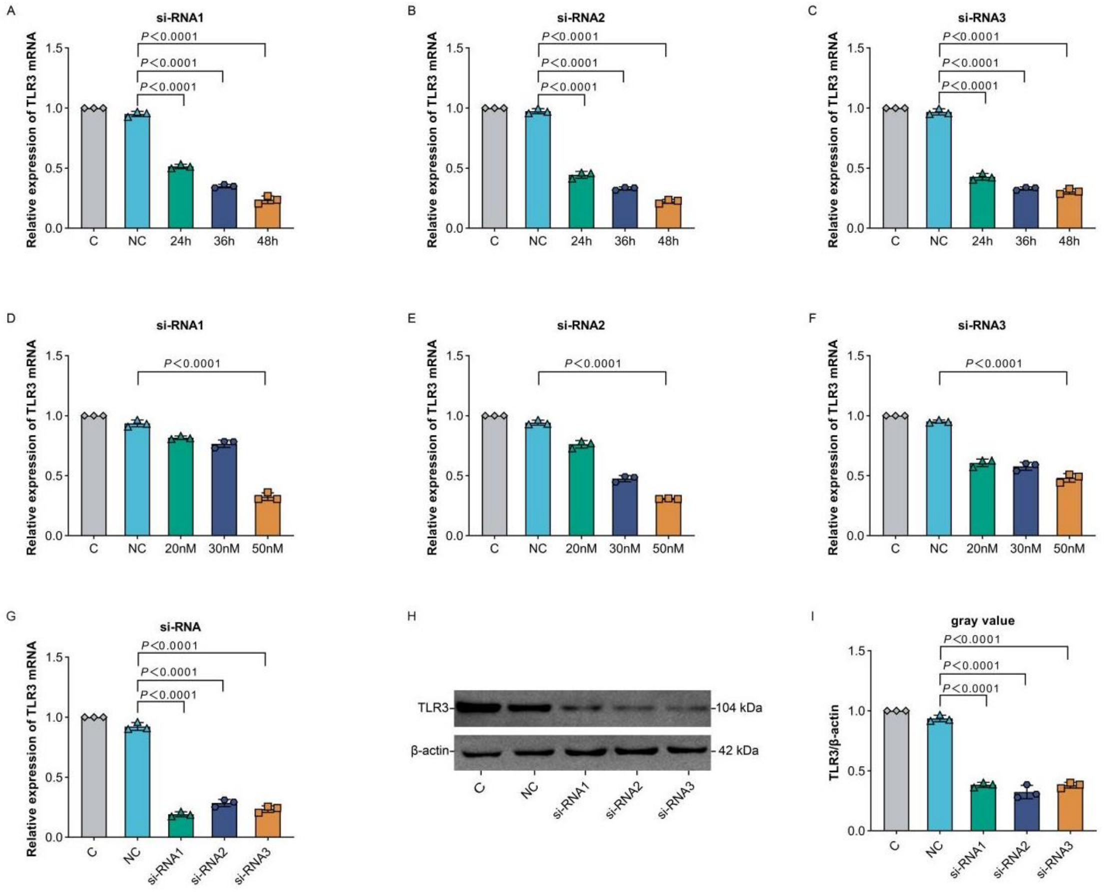


**Figure S2. Evaluation of siRNA's temporal, concentration, and sequence efficacy.**
(A-C) Effects of different siRNAs on the relative expression of TLR3 mRNA under different action time.
(D-F) Effect of different siRNAs on the relative expression of TLR3 mRNA.
(G-I) Effect of different siRNAs on the relative expression of TLR3 protein.
Experiments were performed as three biologically independent experiments, and the mean ± S.D. (n = 3) is shown. P values were determined by non-parametric one-way ANOVA.

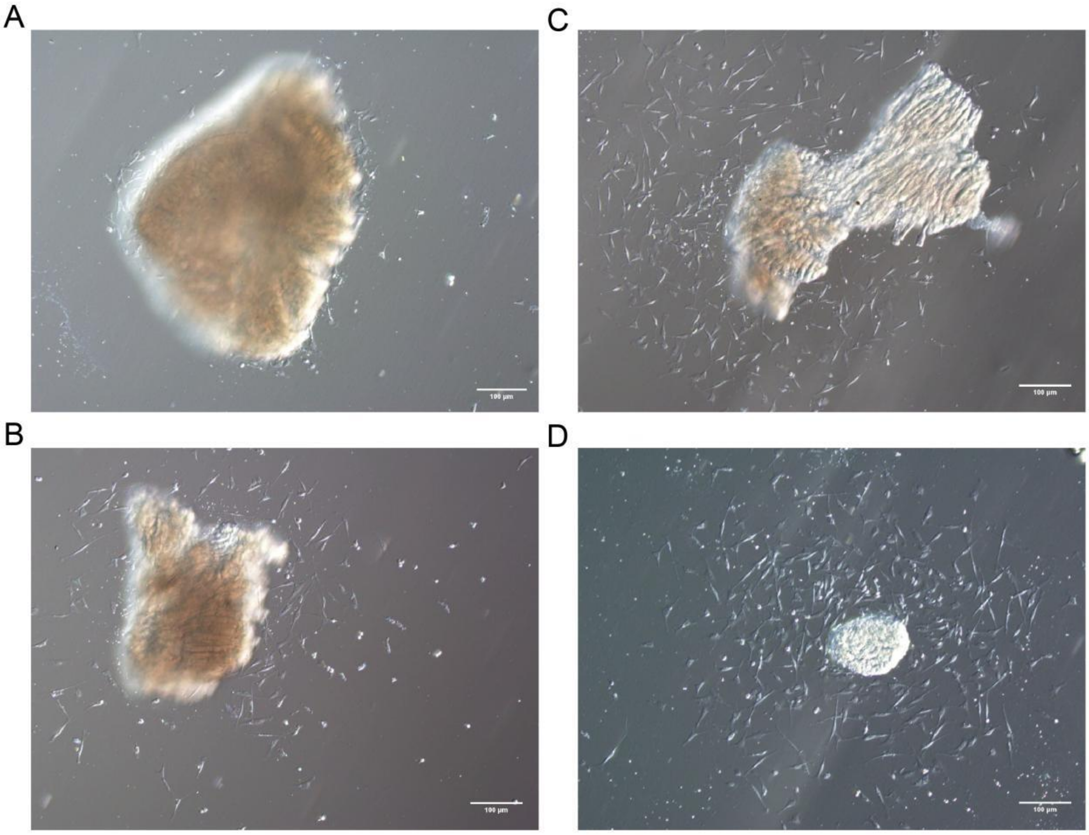


**Figure S3. Monitoring of morphological changes and migration in primary isolated PMVECs over time**

(A) Migration initiation from the tissue block periphery observed at around 6 hours post-culture initiation.
(B-D) Escalation of migration from the tissue block periphery over time, captured under phase-contrast microscopy at various time points. Scale bar = 100 µm

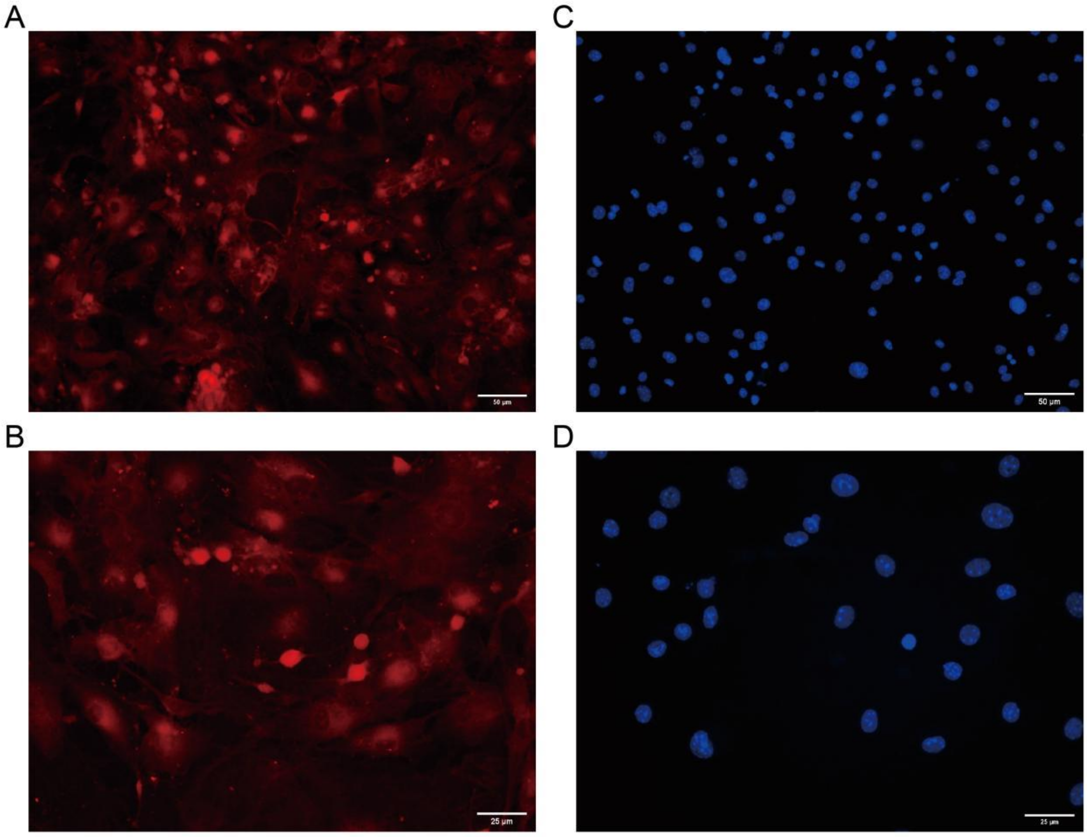


**Figure S4. CD31 Expression in PMVECs Identified through Immunofluorescence Staining**

(A-B) Immunofluorescence identification of CD31 antigen in PMVECs. Images at 200x (A) and 400x (B) magnification depict CD31 expression with red staining.Scale bar =50 μm and 25 μm.

(C-D) Immunofluorescence identification of CD31 antigen in PMVECs. Images at 200x (C) and 400x (D) magnification show nuclei with blue staining by DAPI. Scale bar =50 μm and 25 μm.

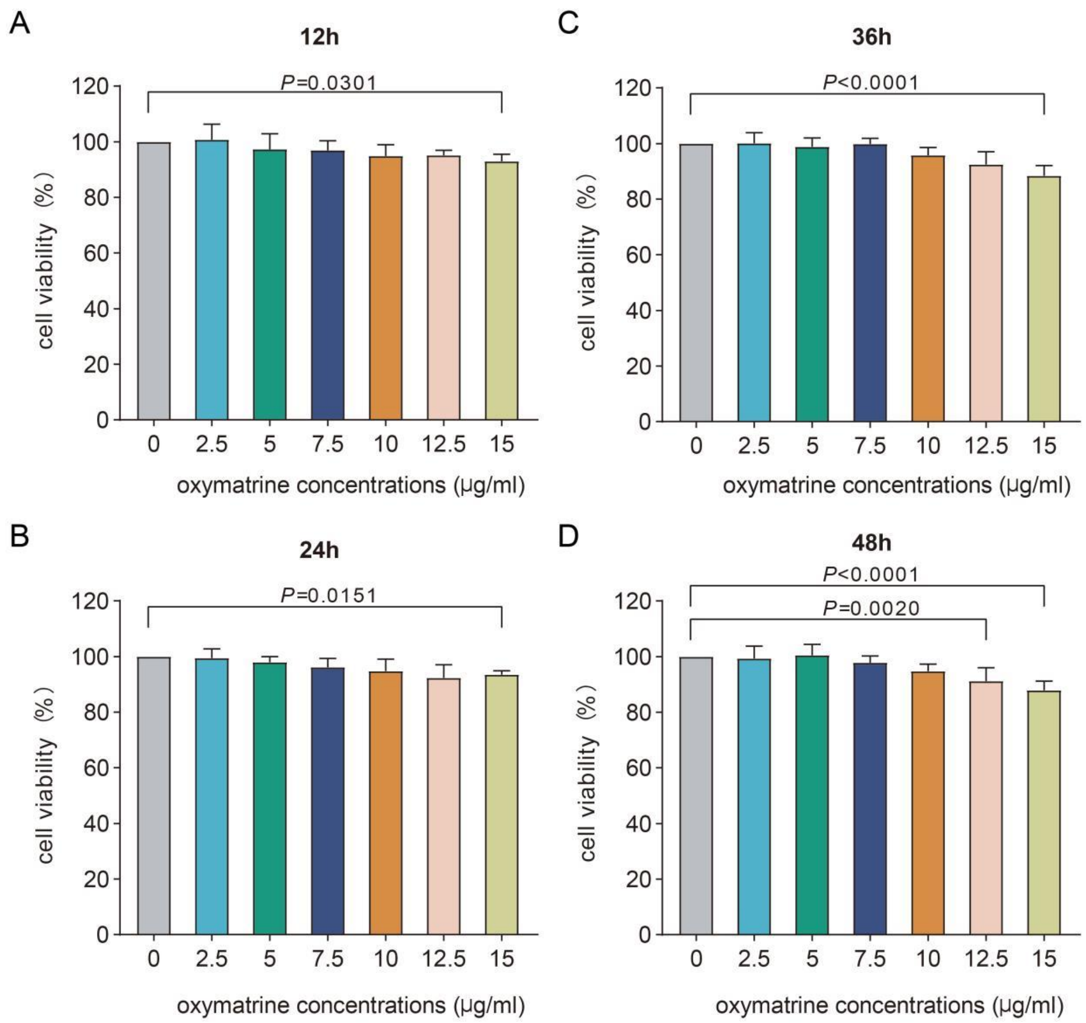


**Figure S5. Cell Viability in PMVECs Following Exposure to a Narrow Concentration Range of OMT Over Various Time Intervals.**

(A-D) This bar graph illustrates the impact of varying concentrations of Oxymatrine (OMT) on the viability of Pulmonary Microvascular Endothelial Cells (PMVECs) across four distinct time intervals: 12h (A), 24h (B), 36h (C), and 48h (D). The cell viability post-OMT exposure was quantitatively evaluated using the CCK-8 assay.

Experiments were performed in triplicate, and the mean ± S.D. (n = 3) is shown. P values were determined by non-parametric one-way ANOVA.
